# The knowledge and perceptions of the first year medical students of an International University on family planning and emergency contraception in Nicosia (TRNC)

**DOI:** 10.1186/s12905-018-0641-x

**Published:** 2018-09-15

**Authors:** Ozen Asut, Ovgu Ozenli, Gizem Gur, Elif Deliceo, Buse Cagin, Okan Korun, Ozgur Turk, Songul Vaizoglu, Sanda Cali

**Affiliations:** 1 Near East University Faculty of Medicine Department of Public Health, Nicosia, Turkish Republic of Northern Cyprus; 2 Anadolu University Medical School, Eskisehir, Turkey; 3 Ministry of Health, Ipsala, Turkey; 4 Burhan Nalbantoglu Hospital, Nicosia, Turkish Republic of Northern Cyprus; 5 Private sector, Nicosia, Turkish Republic of Northern Cyprus; 6 Dr. Suat Gunsel Kyrenia University Hospital, Kyrenia, Turkish Republic of Northern Cyprus

**Keywords:** Family planning, Emergency contraception, Medical students, Knowledge, Perception

## Abstract

**Background:**

Informing the individuals on family planning including emergency contraception is a significant step for preventing unintended pregnancies. Although there is a number of studies on family planning and emergency contraception globally and in Turkey, no such data are available in the Turkish Republic of Northern Cyprus. The objective of this study was to evaluate the knowledge and perceptions on family planning and emergency contraception of the first year students of an international medical school in Nicosia, Northern Cyprus and to increase awareness for developing new policies on the issue.

**Methods:**

The data of this cross-sectional study were collected in February 2016 by a questionnaire of 36 questions. Of the 229 students, 189 (82.5%) completed the questionnaire. The data were evaluated by SPSS 18.0 statistical program. The differences of variables were evaluated by Chi square test, *p* < 0.05 being accepted as significant.

**Results:**

The distribution of participants from 23 countries according to nationality revealed three leading countries: Nigeria, Turkey and Syria. Of the students, 53.6% knew the definition of family planning. The sources of information were mainly school, the internet and media, with a total of 60.9% of the participants who stated having prior information on the subject.

Awareness of contraceptive methods was indicated by more than 90% and emergency contraception by 66.1% of the participants. However, the students were unable to differentiate between modern and traditional family planning methods; 85.6% did not have knowledge of the most effective period for emergency contraception and 63.1%, of the definition of emergency contraceptive pills.

**Conclusions:**

In conclusion, the knowledge and awareness level of the first year medical students on family planning and emergency contraception was insufficient. Family planning and emergency contraception education should be provided for the students at the first year of all faculties as well as medical schools and relevant programs should be included in the curricula of medical education.

**Electronic supplementary material:**

The online version of this article (10.1186/s12905-018-0641-x) contains supplementary material, which is available to authorized users.

## Background

Information provided by the health sector to the individuals in a community on the family planning (FP) concept and methods is a significant first step for the use of appropriate contraceptive methods. For assessing the success of family planning services,contraceptive prevalence rate is the measure used most frequently [1].

The increase in the use of contraceptive methods has decreased maternal and child mortality, and also has influenced the education and economic status of women [2–5]. However, only 32% of married women were currently using a modern contraceptive method, according to a survey of 52 developing countries [2].

Emergency contraception (EC) is a practical prevention method of pregnancy available for sexually active young people. Emergency contraception (EC) is the general term for defining prevention methods of pregnancy in the first few days after sexual intercourse to be applied after unplanned, unprotected or inadequately protected sex, forced sexual interactions or sexual assault as well as contraceptive failures. According to ICEC guidelines, emergency contraceptive pills (ECPs) are indicated for EC by three ECP regimens [6]: levonorgestrel 1.5 mg, ulipristal acetate 30 mg, mifepristone 10–25 mg.

Some types of contraceptive pills can also be used as EC (known as the “Yuzpe regimen”).

The World Health Organization proposes the use of contraceptive pills, such as progesteron preparations (Levonorgestrel) and post-coital intra- uterine device (IUD) insertion for EC.

Young people and university students among them constitute an important populaton group under the risk of unmet needs regarding contraception. Unplanned pregnancies amongst students at higher education institutions influence students’ academic success. Studies in the United States of America (USA) indicate that 80% of female students in higher education are sexually active. Female students are under the risk of unplanned pregnancies as a result of ineffective or non-use of contraceptives. This may result in failure to complete their education, may prevent sustaining employment and making independent marital decisions [7].

Students of 18 to 24 years of age reveal high rates of unplanned pregnancies. The lack of knowledge concerning contraceptive use is one of the major causes of unplanned pregnancies, which result in significant health and social problems. The unplanned pregnancies may occur as a result of not utilizing contraceptive methods or due to the failure of the contraceptive methods used [7, 8].

The Student Health Survey of Finland in 2004 revealed that 80% of the students were sexually active. Of the students, 65% of males and 79% of females declared using contraception. The most popular methods were hormonal contraception for women and condom for men. EC was generally preferred in case of condom failure. One third of the students had not used contraception but most of the students used reliable methods and EC when needed [9].

The Turkish Republic of Northern Cyprus (TRNC) located in Northern Cyprus is a country with a number universities accepting students from all over the world, mostly from the middle income countries of the Middle East Asia and Africa. Hence, family planning including emergency contraception use and access should be considered an important issue of public health among these students separated from their families and homelands.

The family planning methods available for use in the TRNC include oral contraceptives, male condoms, IUDs and injectable hormones. On the other hand, Ulipristal (Ella) and theYuzpe regimen, as well as IUDs are available for EC in the TRNC. These medications are not covered by the social security systems in the country; thus, the individuals have to pay the expenses of the drugs or devices out of pockets. Although there is a number of studies on family planning and emergency contraception globally and in Turkey as well, no such data are available in the Turkish Republic of Northern Cyprus (TRNC) [10–12].

The objective of this study was to evaluate the knowledge and perceptions of the first year students of an international medical school (Near East University Faculty of Medicine- NEUFM) in the TRNC on family planning (FP) and emergency contraception (EC) and to increase awareness on the issue for developing new policies and educational programs, including the medical curriculum.

## Methods

### Study setting and design

This study was conducted at the NEUFM in Nicosia, TRNC during February 2016. Students of this University come from a number of countries of the world with differing socio-economic conditions, mainly from the Middle East and Africa. The study was planned as a cross-sectional survey.

### Source population and sampling technique

The universe of the survey was the first year students of the English Program of the NEUFM. The number of students of this Program was 229. The total 229 students were planned to be the study group without selecting a sample.

### Data collection method

A self-administered questionnaire of 36 questions designed by the researchers in English and Turkish was administered to the students in February 2016. Language preference was left to the students. The Turkish students responded to the questionnaire mainly in Turkish and the other students in English (English and Turkish questionnaires are included as Additional files 1 and 2).

Anonymity and confidentiality were assured through the introduction section of the questionnaire where the participants were informed about the scientific nature and purpose of the study and their informed consents were obtained written and verbally.

Terms and definitions:

- **Knowledge**: Knowing the definitions of FP and EC and the purpose of EC, knowing the most effective time period for EC use.
- **Awareness**: Having heard about FP and EC, and their methods and indications.
- **Data analysis**: The data were evaluated by SPSS 18.0 statistical program. For the parameters analysed, descriptive statistics (frequency distribution, means, medians and standard deviations) were determined and marginal and cross tables were created. The differences of variables between the groups were evaluated by Chi-square test. For the statistical analyses, *p* < 0.05 was accepted as significant.
- **Independent variables** included age, gender, marital status, nationality, the longest place of residence until the age of 12, education of parents, current sexual activity status, the age of first sexual intercourse, source of information on FP.
- **Dependent variables** included the knowledge of the definition of FP, awareness of methods of FP; use of FP methods; the knowledge of the definition and purpose of EC; awareness of methods and indications of EC; the knowledge of the most effective time period for EC use; use of EC methods.

## Results

NEUFM English Medical Program first year students consisted of 229 persons, all of whom were targeted to be included in the study. Of the students, 191students (83.4%) could be accessed because the rest of the students were not attending school during the study period. Excluding two refusals, the survey group was 189 students and hence, the coverage rate was 82.5%. The socio-demographic characteristics of the participants, their nationalities and country of residence until age 12 are presented in Table 1. Of the participants, 48.7% were females and 51.3% males. The age range of the participants was 16–27, with mean and median ages as 19 each.

**Table 1:** Socio-demographic characteristics of English Medical Program first year students of NEU, Northern Cyprus, 2016

Socio-demographic Features					
Age	Mean:19.43 ± Standard Deviation 1.75				
	Median: 19.0				
	Minimum-Maximum: 16–27				
Gender	Male		Female		Total
	*n*	%	*n*	%	
	92	48.7	97	51.3	189
Marital status	Married		Single		Total
	*n*	%	*n*	%	
	4	2.1	183	97.9	187
Country	Nationality		Country of residence until age 12		
	*n*	%	*n*	%	
Nigeria	49	26.5	49	26.5	
TR	31	16.8	33	17.8	
Syria	23	12.4	11	5.9	
Jordan	21	11.4	16	8.6	
TRNC	15	8.1	13	7.0	
Palestine	8	4.3	8	4.3	
Lebanon		3.8	6	3.2	
Libya	5	2.7	5	2.7	
Saudi Arabia	3	1.6	16	8.6	
Iran	3	1.6	2	1.1	
Iraq	3	1.6	3	1.6	
UK	2	1.1	1	0.5	
Egypt	2	1.1	1	0.5	
Russia	2	1.1	1	0.5	
Kuwait	2	1.1	3	1.6	
India	2	1.1	0	–	
UAE	1	0.5	12	6.5	
Somalia	1	0.5	0	–	
Kenya	1	0.5	1	0.5	
Canada	1	0.5	0	–	
Zimbabwe	1	0.5	1	0.5	
Sweden	1	0.5	1	0.5	
Sudan	1	0.5	0	–	
USA	0	–	1	0.5	
Oman	0	–	1	0.5	
Total	185	100.0	185	100.0	

The study participants were from 23 different countries. Most of the participants were from Nigeria (26.5%), Turkey (16.8%), Syria (12.4%), Jordan (11.4) and TRNC (8.1%). Citizens of TRNC were 15 students comprising 8.1% of the participants. The international status of NEU and the pursuit of medical education in English provided the opportunity for the multinational character of the study.

The results regarding the knowledge and awareness of the participants on FP and EC are presented in Table 2. FP was correctly defined by only 53.6% of the students. Of the total, 60.9% (112 students) stated they were informed about family planning, with 53.9% for males and 67.4% for females, and the difference of the two genders was not significant (*p* = 0.06). The education of the father was found influential on the knowledge of the definition of FP, with a statistically significant difference in favor of university educated fathers.

**Table 2:** The knowledge and awareness on family planning and emergency contraception concepts and methods of English Medical Program first year students of NEU, Northern Cyprus, 2016 (*N* = 189)

		Number	Percent
Family planning			
Knowledge of definition (*n* = 181)	Correct	97	53.6
	Incorrect	84	46.4
Having prior information on FP (*n* = 184)	Informed	112	60.9
	Not informed	72	39.1
Source of information (*n* = 111)^a^	School	65	58.6
	Internet	46	41.4
	Media	36	32.4
	Parents, relatives	34	30.6
	Television	32	28.8
	Health professionals	21	18.9
	Friends	17	15.3
	Pharmacy	2	1.8
Awareness of traditional/natural contraceptive methods (*n* = 189)^b^	Withdrawal	48	25.4
	Breast-feeding	60	31.8
	Periodic abstinence	69	36.5
Awareness of 3 effective traditional methods (*n* = 189)	All methods	14	7.4
	Some methods	175	92.6
Awareness of modern methods^b^ (*n* = 189)	Male condom	156	82.5
	Oral contraceptive pill	160	84.7
	Diaphragm	161	85.2
	Female condom	166	87.8
	Implant	171	90.5
	Combined vaginal ring	173	91.5
	Depo hormone injection	176	93.1
	Spermicide	177	93.7
	Female surgical sterilization	177	93.7
	Male surgical sterilization	178	94.2
	Combined patch	179	94.7
	IUD	182	96.3
	Cervical cap	186	98.4
Emergency Contraception			
Knowledge of definition (*n* = 171)	Correct	113	66.1
	Incorrect	58	33.9
Knowledge of purpose (*n* = 176)	Right	113	64.2
	Wrong	63	35.8
Awareness of methods (*n* = 189)	ECPs	94	49.7
	Intra-uterine device	9	4.8
Awareness of all EC methods (*n* = 173)	Complete	1	0.6
	Not complete	172	99.4

^*a*^ *Each row percentage was taken over the 111 respondents of the question*

^*b*^ *Each row percentage was taken over the 189 respondents of the question*

The family planning methods were investigated by a list of 16 FP methods, inquiring to mark the methods as either modern or traditional. Most of the students were aware of the modern contraception methods. The modern methods the students were most aware of were cervical cap (98.4%), IUD (96.3% and combined patch (94.7%). Regarding the effective traditional/natural contraceptive methods, 9.5% had awarenessof all the three effective methods (withdrawal, periodic abstinence and lactational amenorrhea). On the other hand, 15.3% of the students described the oral contraceptive pill and 17.5% the male condom as traditional methods.

The definition of EC was correctly indicated by 66.1% of the participants and its purpose by 64.2%. However, the awareness of EC methods was much lower with 49.7% for ECPs and 4.8% for IUDs. The findings on EC methods are shown in Table 3. The best known EC method was the ECPs. The indications of EC were mostly stated as unprotected sex (58.9%) and unintended pregnancy (53.1%). Less than one fifth of the participants (18.3%) indicated that EC is used in case ofcontraceptive failure. Of the participants, 45.1% stated EC as a method of FP. Most of the participants (85.6%) did not have correct knowledge of the most effective period for EC, which was found to be better known by men but the gender difference was not statistically significant. About two thirds of the participants (68.0%) were not informed about the ECPs compared to 17.9% with correct knowledge. The results were similar for both genders. There was only one person who stated both of the EC methods correctly (the ECPs and IUDs).

**Table 3:** The knowledge on most effective time period for and indications of EC and definition of ECPs of English Medical Program first year students of NEU, Northern Cyprus, 2016 (*N* = 189)

	Number	Percent
Indications of emergency contraception (*n* = 175)^a^		
Unprotected sexual intercourse	103	58.9
Unintended pregnancy	93	53.1
Sexual assault	86	49.1
As a method of family planning	79	45.1
Unsuccessful contraception	32	18.3
Knowledge of the most effective time period for emergency contraception (*n* = 167)		
Correct	24	14.4
Incorrect	143	85.6
Knowledge of the defininiton of ECPs (*n* = 184)		
Correct^b^	33	17.9
Incorrect	9	4.9
Insufficient	26	14.1
No knowledge	116	63.0

^a^ *Row percentage over 175*

^*b*^ *The knowledge of the definition of ECPs was determined by the open ended question in the questionnaire asking the definition of ECPs. The replies were evaluated according to the WHO criteria of ECPs as correct, insufficient and incorrect*

The knowledge of the participants about the provision of emergency contraception devices (IUD) and their adverse effects is presented in Table 4. About one third of the participants of the survey (32.4%) conveyed the opinion that EC methods have side effects, infertility (27.4%) and hormonal disorders (19.6%) being the most frequently indicated of these. About 35% of the students were of the opinion that hospitals are the premises IUD is provided for EC. Table 5 shows the evaluation of the responses of the participants to some statements on FP and EC. Half of the participants (50.8%) shared the opinion that EC did not increase the frequency of unprotected sexual relations. The findings of students from different countries were similar. More than half of the participants (59.3%) were of the opinion that EC reduces abortion frequency. Half of the participants stated that people are embarrassed while buying ECPs from the pharmacy, an opinion which was significantly higher among Asian country citizens.

**Table 4:** The status on the information about the provision of emergency contraception devices (IUD) and their adverse effects of English Medical Program first year students of NEU, Northern Cyprus, 2016 (*N* = 189)

	Number	Percent
Localization of IUD insertion process (*n* = 175)^a^		
Hospital	61	34.9
Family health center	13	7.4
Pharmacy	9	5.1
Private office	7	4
Health center	4	2.3
Home	3	1.7
Side effects of emergency contraception (*n* = 182)		
Adverse effects exist	59	32.4
No adverse effects	46	25.3
Don’t know	77	42.3
Adverse effects of emergency contraception as expressed by the participants (*n* = 51)^b^		
Infertility	14	27.5
Hormonal disorder	10	19.6
Bleeding	8	15.7
Injury to uterine wall	7	13.7
Menstrual disorders	7	13.7
Constitutional syptoms	6	11.8
Infection	2	3.9
Weight gain	2	3.9
Cancer	1	2.0
Liver injury	1	2.0
Depression	1	2.0
Death	1	2.0

^*a*^ *Each row percentage is over 175 persons who responded to this question*

^*b*^ *Each row percentage is over 51 persons who responded to this question*

**Table 5:** Responses to some statements on family planning and emergency contraception of English Medical Program first year students of NEU, Northern Cyprus, 2016 (*N* = 189)

	Right		Wrong	No knowledge		
	*n*%		*n*%	*n*		%
Emergency contraception increases the frequency of unprotected sexual intercourse (*n* = 177) (W)	42	23.7	90	50.9	45	25.4
Emergency contraception decreases the abortion rate due to unintended pregnancies (*n* = 177) (R)	105	59.3	16	9.0	56	31.6
People are embarrassed to buy ECPs from pharmacies (*n* = 175) (R)	88	50.3	25	14.3	62	35.4
Abortion is a method of emergency contraception (*n* = 177) (W)	36	20.3	45	25.4	96	54.2
Family planning is mainly women’s responsibility (*n* = 176) (W)	130	73.9	30	17.1	16	9.1
Modern methods are more effective than traditional methods (*n* = 176) (R)	144	81.8	8	4.6	24	13.6

As is presented in Table 6, participants who stated having a previous sexual activity comprised 25.4% of the students. The mean age of first sexual interaction was 17 ± 2.0 with a minimum age of 12 and a maximum of 23. Some form of FP method was used at first sexual relationship by 52.6% of the students who acknowledged having previous sex experience and the method preferred most frequently was the condom by 71.4%. Five (16.7%) of the students who stated having prior sexual experience had used ECPs. Of those who experienced a previous sexual relation, 46.5% knew the definition of ECPs, whereas the percentage was 29.6% for those not having a previous sex history. The difference of the two groups was found to be statistically significant, the knowledge of the ECP definition being higher in those who had prior sexual experience and highest in the participants who received FP information from health professionals, the media and the internet.

**Table 6:** The status regarding sexual activity and family planning method use of English Medical Program first year students of NEU, Northern Cyprus, 2016 (*N* = 189)

	Number	Percent
Sexual activity experience (*n* = 169)		
Yes	43	25.4
No	126	74.6
Age of first sexual interaction (*n* = 37)		
< 14	3	8.1
14	2	5.4
15	4	10.8
16	4	10.8
17	7	18.9
18	9	24.3
19	3	8.1
≥ 20	5	13.5
Mean: 17 ± SD2.05 Median: 17 Minimum - Maximum: 12–23		
Use of family planning method at first sexual intercourse (*n* = 38)		
Yes	20	52.6
No	18	47.4
Family planning method used at first sexual relation (*n* = 28)^a^		
Condom	20	71.4
Withdrawal	6	21.4
Oral contraceptive pill	2	7.1
Spermicide	1	3.6
ECPs	1	3.6
Current regular sexual activity (*n* = 46)^b^		
Yes	19	41.3
No	27	58.7

^*a*^ *Each row percentage was calculated over 28 persons*

^*b*^ *Current regular sexual activity was determined according to the reply to the relevant question*

The distribution of the knowledge, awareness and behavior of the participants on FP methods and EC in relation to some socio-demographic features including age, nationality and gender are given in Table 7.

**Table 7:** Distribution of correct knowledge of participants on family planning and emergency contraception in relation to some socio-demographic features among English Medical Program first year students of NEU, Northern Cyprus, 2016 (*N* = 189)

		Correct knowledge^a^			
		*n*	%	χ^2^	*p*
Definition of family planing					
Gender	Male	48	53.9	3.5	0.062
	Female	64	67.4		
Age group	< 20	51	44.0	8.5	**0.004**
	≥20	45	66.2		
Nationality^a^	TRNC	3	20.0	44.8	0.000
	TR	7	22.6		
	African countries	51	85.0		
	Asian countries	34	46.6		
	European/American countries	2	33.3		
Country of residence until age 12^a^	TRNC	3	23.1	51.7	**0.000**
	TR	7	21.2		
	African countries	51	89.5		
	Asian countries	35	44.9		
	European/American countries	1	25.0		
Education of father	High school and under	13	34.2	5.6	**0.018**
	University	84	55.6		
Definition of emergency contracep-tion					
Gender	Male	52	56.5	0.8	0.372
	Female	61	62.9		
Age group	< 20	64	55.2	2.2	0.143
	≥20	45	66.2		
Nationality^a^	TRNC	14	93.3	25.1	**0.000**
	TR	25	80.6		
	African countries	38	63.3		
	Asian countries	30	41.1		
	European/American countries	5	83.3		
Country of residence until age 12^b^	TRNC	12	92.3	20.3	**0.000**
	TR	26	78.8		
	African countries	37	64.9		
	Asian countries	34	43.6		
	European/ American countries	3	75.0		

^*a*^ *Correct knowledge of family planning and emergency contraception were evaluated separately according to the relevant questions about the definitions of FP and EC in the questionnaire*

^*b*^ *In the analyses, TRNC, TR, European and American countries, were grouped together as one group*

The numbers in bold show statistical significance

The main sources of information on FP were school and the internet. While more than half of men (58.3%) indicated the internet as the source of their information, school was the main source for 66.7% of the women. Only 18.9% of the students expressed health professionals as the information source for FP.

The distribution of knowledge and behavior of the participants on FP and EC according to their gender and nationality are presented in Table 8. Receiving FP information from the internet was found to be higher in men in comparison to women, the difference being statistically significant (*p* = 0.002). On the other hand, receiving FP information from school was higher in women in comparison to men at a statistically significant level (*p* = 0.047).

**Table 8:** The distribution of knowledge and behavior of participants on family planning and emergency contraception according to gender and nationality among English Medical Program first year students of NEU, Northern Cyprus, 2016 (*N* = 189)

Gender								
	Male			Female				
	*n*		%	*n*		%	χ^2^	*p*
Being informed on FP	48		53.9	64		67.4	3.5	0.062
Receiving FP information from internet	28		58.3	18		28.6	9.9	**0.002**
Receiving FP information from school	23		47.9	42		66.7	3.9	**0.047**
Awareness of three effective natural/traditional contraceptive methods	–		–	14		17.9	13.9	**0.000**
Use of FP methods by close contact persons	35		39.8	44		46.8	0.9	0.339
Use of condomby close contact persons	25		75.8	14		31.8	14.6	**0.000**
Knowledge of purpose of emergency contraception	52		61.2	61		67	1.2	0.547
Knowledge of most effective period for emergency contraception	13		16	11		12.8	0.5	0.909
Information of ECPs	15		16.9	18		18.9	1.1	0.782
Status of age of first sexual interaction being under or equal to 17 years	16		57.1	4		44.4	F^a^	0.703
Useof contraceptive method at first sexual intercourse	12		44.4	8		72.7	2.5	0.113
Current regular sexual relationship	13		40.6	6		42.9	0.02	0.887
Nationality								
	TRNC-TR- Other		African countries		Asian countries			
	*n*	%	*n*	%	*n*	%	χ^2^	*p*
Being informed on FP	17	32.7	48	82.8	44	62.9	29.0	**0.000**
Awareness of three effective natural/traditional contraceptive methods	1	2.4	12	24	1	1.9	^*^	0.213
Use of FP methods in the vicinity	17	32.7	28	49.1	30	43.5	3.1	0.213
Knowledge of purpose of emergency contraception	37	72.5	41	74.5	33	50	10.5	**0.033**
Age of first sexual interaction being under or equal to 17 years (*n* = 35)	12	60	2	40	5	50	F^*^	0.551
Use of contraceptive method at first sexual intercourse (*n* = 37)	13	65	4	80	3	25	F^*^	0.41
Current regular sexual relationship (*n* = 43)	9	42.9	3	37.5	6	42.9	F^*^	0.981
Knowledge of decrease in abortion frequency by EC	2	4.1	3	5.5	11	15.9	12.1	**0.016**
Knowing abortion is not a method of EC	18	36.7	7	12.7	10	14.5	13.2	**0.010**

^a^ *Analysed by Fisher’s exact test*

The numbers in bold show statistical significance

Regarding the responsibility of the two genders on FP, 17.1% of the male participants and 17.0% of the female participants stated that family planning is essentially women’s responsibility, whereas 70.7% of males and 76.6% of females responded on the contrary; that FP is not primarily women’s responsibility. There was no significant difference between genders regarding the idea of assigning primarily women for FP (*p* **=** 0.4).

## Dıscussıon

The resultsof this study revealed that more than half of the students had previous information on FP. The majority of the participants correctly responded that modern methods are superior to traditional methods. However, most of the responders who stated being informed about FP could not differentiate between modern and traditional methods.

Regarding knowledge on FP, studies among high school students in Brazil and college students in India, revealed that the majority of the students were more informed about FP, in comparison to our study [13, 14]. In our study, the differences among country groups regarding some of the findings were found significant, such as being informed on FP, knowing the purpose of EC and knowing the decrease in abortion frequency by EC. Participants from African countries had knowledge of FP and purpose of EC more than others. Members of Asian countries on the other hand, were informed more than others of the decrease in abortion frequency as a consequence of EC. However, the number of students are insufficient for inferring definite conclusions about the situation in countries according to these findings.The main sources of information on FP were school for women and the internet for men. The level of the role of health professionals on FP education was found to be lower (less than one fifth of the participants). Our results differ from two studies in India, where the major sources of information were the media [14] and friends [15].

The findings on the knowledge of the definition and purpose of EC are similar to a study performed in a university of Ethiopia [16]. Awareness of IUD as a method of EC was very low. These results are similar to a study in India, where the best known EC method was the ECPs and the knowledge of the use of IUD was low [14]. In general, the awareness and use of IUD as a method of EC is a slow process globally, requiring education and publicity by the national health services [6, 17]. In our study, the indications of EC were stated mostly as unprotected sex, unintended pregnancy and sexual assault. Less than one fifth of the participants were aware that EC is also used in case of contraceptive failure, contrary to a study in a university of Ethiopia, where it was the best known indication of EC [18]. In a study performed in South Africa among university students, the indication of EC stated most by the participants was sexual assault [10], which ranked third in our study.

Most of the participants of the current study did not have correct knowledge of the most effective period for EC, similar to studies on university students of South Africa and on adolescents and young people in India [10, 14]. On the other hand, a study of the university students in North West Ethiopia has shown that the majority of the participants had correct knowledge of the most effective period for EC [18]. In our study, the most effective period of EC was found to be better known by men but the gender difference was not significant. This finding differs from the results of a study among the university students of Ghana, where women had better knowledge on this issue [19].

One thirds of our participants stated that EC methods have side effects, mostly infertility and hormonal disorders. Infertility as an adverse effect of EC has been mentioned in studies among university students in Ethiopia and South Africa [10, 18]. A study in India also revealed that a great majority indicated hormonal disorders as a side effect of EC [14].

One fourths of the participants in our study stated having previous sexual activity, which is similar to the findings of Adis Ababa University female undergraduate students [20]. The mean age of first sexual intercourse of those students with former sexual history was 17, which is similar to the studies of university students of North West and South West Ethiopia, Brazil and also medical school students of India [13, 15, 16, 18]. About half of those participants who had previous sexual activity stated having first sexual intercourse before or equal to age 17. Our findings about age of first sexual intercourse are similar to other country results [13, 15, 16, 18, 21]. The study of Aberdeen University female students revealed a higher level of prior sexual activity than the participants of our study, with 40% [22].

In our study, the majority of the women with previous sexual experience had used an FP method in their first sexual intercourse, the condom being the leading method. This finding is in accordance with the survey carried out among adolescents in Nigeria [23]. The use of contraceptive method at first sexual relation was highest among African country citizens in our study, similar to the results of African country studies like Ethiopia and Nigeria.

The level of knowledge of the students on EC concept and methods was low in our study. Similar findings on this issue were determined in other studies such as the studies among university students in South Africa and Ghana, adolescents and young people in India and high school students in Brazil [10, 13, 14, 19]. On the contrary, EC knowledge was found to be high in some studies like university students in Nigeria, women in Sweden and the broad EC survey in Camerun [24–26]. Similarly, the study among female undergraduate students in Adis Ababa University revealed the knowledge of EC as 84.2% [16].

A study on EC conducted among first year medical students in India also showed higher awareness of EC than the results of our study. All the students were aware of the existence of emergency contraception, also knowing the correct time limit. But there was lack of knowledge about other aspects of EC. First year medical students were evaluated as no different than other under graduates regarding EC, other than awareness of its existence and correct timing of use [27], which is also relevant for our study.

The findings of our study, as well as other similar country research indicate that the knowledge and perceptions on FP and EC may generally be related to the socio-economic development and educational status of the communities.

The majority of the students in this study were from Nigeria, Turkey, Syria, Jordan and TRNC. With the exception of TRNC, these countries are middle income countries (Turkey being classified as upper middle and the other three as lower middle income countries). Only TRNC has a per capita gross national income (GNI) in the range of high income countries [28, 29]. TRNC had a gross domestic product (GDP) per capita of 13,737 USA$ in the year 2015 [30].

For a comparison of our survey with the countries of origin of our participants, the findings of Demographic and Health Surveys (DHS) of three of these countries will be mentioned. DHS of 2013 of Nigeria reveals that age of first sexual intecourse is 17.9 in women of 20–24 age group and 20.9 in men of 25–29 age group. Knowledge of any method is 95% in men and 85% in women; of modern methods 94% for men and 84% for women. Contraceptive pill is the best known modern method by 71% of women and male condom by 91% of men. However, any contraceptive method use prevalence is 15% and modern method use 10% in general [31].

According to the 2012 DHS in Jordan, the knowledge of at least one contraceptive method among ever-married women was high. Of the women, prevalence of any family planning method is 60% and use of modern methods is 42%. The private sector provides 56% of contraceptives and the public 44%. Governmental health centers provide 23% of contraceptive services [32].

In Turkey, DHS 2014 has revealed that any contraceptive method prevalence has increased, starting from 2003, rising up to 74% in 2013. Modern method preference shifted in a 25-year period from 31 to 47% in 2013. The increase in use of modern methods resulted in a decline of traditional methods with the exception of withdrawal, which preserved its high level [1].

The general level of knowledge and perceptions of the participants of our study are similar to the overall characteristics of the three countries, reflecting the countries of their origin, pointing out to a need of additional education and free provision of drugs and devices for success in changes in knowledge, attitudes and behaviors. Different from approaches for the general population, special measures are needed for higher education populations about FP and EC for healthy sexual behaviors and avoiding unintentional pregnancies, which may end up with health and other social implications. Medical students may be a starting point among university students.

National and international action is needed for developing programs for university students and other young people to promote healthy lives and prevent related problems. These programs should involve students and the faculty staff as well and should be accessible and sustainable.

## The Limitatons of the study

Since there were questions on sexual issues, the responses may be biased. Anonymity was assured to minimize this negative influence. Some of the questions aiming the evaluation of knowledge of the students on FP-EC may not have yielded precise answers because of the wording of the questions and evaluation based on exclusively participant responses. Some responses may have been hinted in the succeeding questions. Cervical cap, IUDs and the combined patch were found to be the top well-known modern methods, which might be a reflection of common sense rather than specific knowledge of the methods. The authors are in the opinion that open-ended questions might have been more appropriate for this topic.

Another limitation of the study was the coverage of only first year medical students. Consequently, the results of this study cannot be generalized to university students in the TRNC. Additional research is needed to attain definite conclusions about the knowledge and perceptions of medical students and other university students of TRNC on the issue.

## Conclusions

In conclusion, the knowledge and awareness level of the first year medical students on FP and EC were deemed insufficient according to the results of the current study. The condom for FP and the ECPs for EC were the methods used most by the participants who were experiencing sexual activity. The role of the health profession on informing the students about FP and EC was found to be limited, which preferably should be the primary method of enlightening young people on the issue.

The contraceptive method use of adolescents and young people differs significantly from adults and married couples. The difference is due to educational, developmental, social and psychological factors. For this reason, the adolescents need a dynamic education on FP and EC. Without discriminating by gender, nationality or age, education and counseling should be provided for the first year students of all faculties including medical students, at health centers of university campuses, as well as programs in the currricula of medical schools, preferably starting the first year of education. Additionally, the development of innovative and scientific educational programs and community activities are crucial for informing young people about healthy sex activites. Additional research is needed to develop effective educational programs and counseling for the university students in general and specifically for the medical students in TRNC.

Even if the knowledge and perceptions of the people on FP and EC are at high levels, behaviors may not comply with their educational status. An important issue is the socio-economic conditions of the people, which is a barrier for more positive behaviors in health-related problems. The present status of world populations including university students point out to a need for public services for FP and EC and to a need of additional education for success in changes in knowledge, attitudes and behaviors.

## Additional files

Additional file 1: English questionnaire. (DOCX 17 kb)

Additional file 2: Turkish questionnaire. (DOCX 30 kb)

## Abbrevıatıons

DHS: Demographic and Health Survey
EC: Emergency contraception
FP: Family planning
GDP: Gross domestic product
GNI: Gross national income
IUD: Intra-uterine devices
NEU: Near East University
NEUFM: Near East University Faculty of Medicine
TR: Turkish Republic
TRNC: Turkish Republic of Northern Cyprus
UAE: United Arab Emirates
UK: United Kingdom
USA: United States of America

## References

[1] Hacettepe University Population Studies Institute. Turkey demographic and health survey (TDHS) 2013–2014. Ankara: 2014. Available at: http://ghdx.healthdata.org/record/turkey-demographic-and-health-survey-2013-2014

[2] Westoff CF (2012). Unmet need for modern contraception methods. DHS analytical studies 28.

[3] Ahmed S, Li Q, Liu L, Tsui AO (2012). Maternal deaths averted by contraceptive use: an analysis of 172 countries. Lancet.

[4] Rutstein S, Winter R (2015). Contraception needed to avoid high-fertility-risk births, and maternal and child deaths that would be averted. DHS analytical studies, no.50.

[5] Canning D, Schultz TP (2012). The economic consequences of reproductive health and pl planning. Lancet.

[6] ICEC and FIGO. Emergency Contraceptive pills. P. 5. Available at: http://www.cecinfo.org/custom-content/uploads/2014/01/ICEC_Medical-and-Service-Delivery-Guildelines-English_June-2013.pdf

[7] Coetzee MH, Ngunyulu RN. Assessing the use of contraceptives by female undergraduate students in a selected higher educational institution in Gauteng. Curationis 38(2);1–7. Art. #1535, 7 pages. 10.4102/curationis.v38i2.1535, http://www.scielo.org.za/pdf/cura/v38n2/19.pdf Accessed 3 Jul 2018. 10.4102/curationis.v38i2.1535 PMC6091716 26842088

[8] Hoque ME, Ntsipe T, Mokgatle-Nthabu M. Awareness and practices of contraceptive use among university students in Botswana. SAHARA J 2013 Jun; 10(2): 83–88. Published online 2014 Jan 3. doi: 10.1080/17290376.2013.869649PMCID: PMC3914499, PMID: 24405283https://www.ncbi.nlm.nih.gov/pmc/articles/PMC3914499/Accessed 3 Jul 2018. 10.1080/17290376.2013.869649 PMC3914499 24405283

[9] Virtala A. Family Planning among university students in Finland- TamPub 2007. https://tampub.uta.fi/bitstream/handle/10024/67708/978-951-44-6888-9.pdf?sequence=1 Accessed 3 Jul 2018.

[10] Hoque ME, Ghuman S (2012). Knowledge, practices, and attitudes of EC among female university students in KwaZulu-Natal (South Africa). PLoS One.

[11] Daniels K, Jones J, Abma J. Use of EC among women aged 15–44: United States, 2006–2010. US Department of Health and Human Services, Centers for Disease Control and Prevention, NCHS Data Brief No.112. 2013.

[12] Craig AD, Dehlendorf C, Borrero S (2014). Exploring young adults' contraceptive knowledge and attitudes: disparities by race/ethnicity and age. Womens Health Issues.

[13] Martins LBM, Costa-Paiva L, Osis MJD (2006). Knowledge of contraceptive methods among adolescent students. Rev Saude Publica.

[14] Puri S, Bhatia V, Swami HM, Singh A (2007). Awareness of EC among female college students in Chandigarh, India. Indian J Med Sci.

[15] Aggarwal O, Sharma AK, Chhabra P (2000). Study in sexuality of medical college students in India. J Adolesc Health.

[16] Gashaw BT, Tesso FY, Shiferaw BZ (2015). Factors associated with utilization of EC among female students in Mizan-Tepi University, south West Ethiopia. BMC Res Notes.

[17] Emergency contraception fact sheet. Reviewed February 2018. World Health Organization Media Centre Fact Sheet N244, 2012. http://www.who.int/mediacentre/factsheets/fs244/en/ Accessed 2 Feb 2016.

[18] Wasie B, Belyhun Y, Moges B, Amare B (2012). Effect of emergency oral contraceptive use on condom utilization and sexual risk taking behaviours among university students, Northwest Ethiopia: a cross-sectional study. Wasie et al. BMC Research Notes.

[19] Addo VN, Tagoe-Darko ED (2009). Knowledge, practices, and attitudes regarding EC among students at a university in Ghana. Int J Gynecol Obstet.

[20] Ahmed FA, Moussa KM, Petterson KO, Asamoah BO. Assessing knowledge, attitude, and practice of EC: a cross – sectional study among Ethiopian undergraduate female students. BMC Public Health 2012; 12:110 DOI: 10.1186/1471-2458-12-110. https://bmcpublichealth.biomedcentral.com/articles/10.1186/1471-2458-12-110#Abs1. Accessed 12 Sep2016. 10.1186/1471-2458-12-110 PMC3293041 22321964

[21] Fantahun M, Chala F, Loha M (1995). Knowledge, attitude and practice of family planning among senior high school students in North Gondar. Ethiop Med J.

[22] McCance C, Hall DJ (1972). Sexual behaviour and contraceptive practice of unmarried female undergraduates at Aberdeen University. Br Med J.

[23] Araoye MO, Fakeye OO, Jolayemi ET (1998). Contraceptive method choices among adolescents in a Nigerian tertiary institution. West Afr J Med.

[24] Aziken ME, Okonta PI, Ande ABA (2003). Knowledge and perception of EC among female Nigerian undergraduates. Int Fam Plan Perspect.

[25] Larsson M, Aneblom G, Eurenius K, Westerling R, Tydén T (2006). The adoption of new contraceptives methods- surveys and intervention regarding EC. Acta Obstet Gynecol Scand.

[26] Goulard H, Moreau C, Gilbert F, Job-Spira N, Bajos N (2006). Contraceptive failures and determinants of EC use. Contraception.

[27] Chandna A, Nath J, Dhingra D. Awareness of emergency contraception among 1st year medical students. IJCMR 2016;3(6):1568–1570. ISSN (Online): 2393-915X; (Print): 2454–7379. Available at:https://www.ijcmr.com/uploads/7/7/4/6/77464738/_awareness_of_emergency_contraception_among_1st_year_medical_students.pdf Accessed 1 Apr 2018.

[28] Department of Economic and Social Affairs of the United Nations Secretariat (UN/DESA). World Economic Situation and Prospects 2014. Available at:http://www.un.org/en/development/desa/policy/wesp/wesp_current/2014wesp_country_classification.pdf Accessed 31 Oct 2017.

[29] National Accounts Main Aggregates Database 2015. (Select all countries, "GDP, Per Capita GDP - US Dollars", and 2015 to generate table), United Nations Statistics Division. Available at: https://unstats.un.org/unsd/nationalaccount/madt.asp, https://unstats.un.org/unsd/nationalaccount/pubsDB.asp?pType=3.

[30] Tasiran AC, Ozoglu B. Northern Cyprus economy competitiveness report. Turkish Cypriot Chamber of Commerce, 2017. Available at: http://www.ktto.net/wp-content/uploads/2017/03/KTTO2016-2017-eng.pdf Accessed 31 Oct 2017.

[31] National Population Commission (NPC) [Nigeria] and ICF International. Nigeria Demographic and Health Survey 2013.Abuja, Nigeria, and Rockville, Maryland, USA: NPC and ICF International, 2014. Pp. 59, 90. Available at https://dhsprogram.com/pubs/pdf/FR293/FR293.pdf Accessed: 2Oct 2017).

[32] Department of Statistics [Jordan] and ICF International. Jordan Population and Family Health Survey 2012.Calverton, Maryland, USA: Department of Statistics and ICF International., 2013. P. 69–70. Available at https://dhsprogram.com/pubs/pdf/FR282/FR282.pdf Accessed 2 Oct 2017).

