# Supplementary material for: The knowledge and perceptions of the first year medical students of an International University on family planning and emergency contraception in Nicosia (TRNC)
Source: BMC Womens Health. 2018 Sep 15;18:149. doi: 10.1186/s12905-018-0641-x (PMC6139172; doi:10.1186/s12905-018-0641-x)
Supplement: Supplementary file 1 — English questionnaire. (DOCX 17 kb) [file 12905_2018_641_MOESM1_ESM.docx]

**EVALUATION OF KNOWLEDGE AND BEHAVIOR OF FAMILY PLANNING AND EMERGENCY CONTRACEPTION METHODS AMONG NEAR EAST UNIVERSITY FACULTY OF MEDICINE PHASE 1 STUDENTS**

QUESTIONNAIRE FORM

No: ……..

**Please do not write your name on the questionnaire form.**

**I agree to join the research. □**

**I do not agree to join the research. □**

**A. SOCIODEMOGRAPHIC INFORMATION**

**1. How old are you?** (**Write the age you have completed.**) ……….

**2. What is your gender?** 1)Male 2) Female

**3. Please state your citizenship.** ………………………………

**4. In which country did you reside the most until the age of 12?** 1) TRNC 2) Turkey 3) United Kingdom 4) Syria 5) Other (Please state ...............................................)

**5. What is your marital status?** 1) Married 2) Single 3) Other …………………….

**6. What is your mother's education status?** 1) Never been to school 2) Left the primary school 3) Primary school 4) Secondary and high school 5) University

**7. What is your father's education status?** 1) Never been to school 2) Left the primary school 3) Primary school 4) Secondary and high school 5) University

**8.Who are you living with currently?**

1) With family 2) Alone 3) With partner 4) With friend/s

5) Other (Please state ………………......................)

**B. QUESTIONS ABOUT FAMILY PLANNING METHODS**

**9. Which of the following describes family planning the best?**

1) Family planning means to prevent families from having children.

2) Family planning means to plan material and moral sources for the family.

3) Family planning means allowing people to attain the desired number of children when they want and determine the spacing of pregnancies.

4) Family planning is to get couples to have at least one child.

5) Family planning is helping infertile couples to have children through in vitro fertilization.

**10. Have you had any information about family planning/emergency contraception before?**

1) Yes 2) No **(Go to question No.12)**

**11. Where did you get the information about family planning methods? (You can choose more than one option)**

1) Health care workers

2) Pharmacy

3) Internet

4) Media (Magazine / newspaper / book)

5) Friend

6) Mother, father, relative

7) School

8) TV

9) Other (State: …...………………….)

**12. Which of the followings are modern (M) and which are traditional (T) contraceptive methods? (Indicate as M or T)**

| - Oral contraceptive pill | - Intrauterine device (IUD) |
| --- | --- |
| - Lactation | - Calendar method |
| - Spermicide | - Female sterilization (tube ligation) |
| - Withdrawal | - Male sterilization (vasectomy) |
| - Depot hormone injection | - Male Condom |
| - Combined patch | - Female Condom |
| - Combined vaginal ring | - Diaphragm |
| - Implant | - Cervical cap |

**13. Is there anyone you know who is using contraception?**

1) Yes 2) No **(Go to question No.15)**

**14. Please choose which contraceptive method they are using. (You can choose more than one option.)**

1) Pill 2) Condom 3) IUD 4) Injection 5) Tube ligation 6) Vasectomy 7) Periodic abstinence 8) Withdrawal 9) Other State……………………)

**C. INFORMATION ABOUT EMERGENCY CONTRACEPTION**

**15. What is emergency contraception in your opinion?**

1) A birth control method that needs to be taken every day.

2) A birth control method that can be used after unprotected sex.

3) A method to prevent sexually transmitted diseases.

**16. What is the purpose of emergency contraception?**

1) To end the pregnancy.

2) To prevent pregnancy by inhibiting implantation.

3) To prevent sexually transmitted diseases.

**17. Please choose the emergency contraception methods in the list. (You can choose more than one option)**

1. Condom 2. ECPs 3. Combined oral pill

4. IUD 5. Implant 6. Injectable contraceptive

7. Withdrawal 8. Abortion

**18. In which of the following situations can emergency contraception be used? (You can choose more than one option)**

1) Unprotected sexual intercourse

2) Failure of contraceptive method

3) Sexual assault

4) Unintended pregnancies

5) As a family planning method

**19. Which is the most effective period for emergency contraception?**

1) Before sexual intercourse

2) Within first 24 hours after unprotected sex

3) Within 120 hours (5 days) after unprotected sex

4) Until the next menstruation

**20. Where can you get the emergency contraceptive pills? (You can choose more than one option.)**

1) Family health centers 2) Pharmacies with prescription 3) Pharmacies without prescription

**21. Where you can get intrauterine device insertion? ……………………………………………………………………........**

**22. Do you think emergency contraception has side effects which will affect health negatively? If yes please indicate: ……………………………………………………………………………………………………………………………………………………………………………………**

**23. Does anybody close to you ever need this service?**

1) Yes 2) No

**If yes, please indicate what he / she did: ..............................................................................................................................................................................................................................................................................................................**

**24. What is emergency contraceptive pill? Explain briefly: ...................................................................**

**…………………………………………………………………………………………...............**

**25. Have you ever had sexual intercourse?**

1) Yes

2) No **(Go to question No. 31)**

**26. How old were you when you had your first sexual intercourse? Please indicate: …………….**

**27. Did you / your partner use any methods to prevent pregnancy in your first intercourse?**

1) Yes **(Please indicate the method: …………………………………………………………)**

2) No

**28. Do you currently have a regular sexual life?**

1) Yes 2) No

**29. Have you/ your partner ever used an emergency contraceptive method?**

1) Yes 2) No **(Go to question No. 31)**

**30. Please indicate the method you/ your partner used: ………………………………….**

**D. PLEASE SELECT TRUE (T), FALSE (F), I DON’T KNOW (IDK) FOR THE FOLLOWING STATEMENTS**

| **Questions** | **T** | **F** | **IDK** |
| --- | --- | --- | --- |
| **31. Emergency contraception increases the frequency of unprotected sexual intercourse.** |  |  |  |
| **32. Emergency contraception decreases the frequency of abortions related to unintended pregnancies.** |  |  |  |
| **33. People get embarrassed when buying emergency contraceptive pill from the pharmacy.** |  |  |  |
| **34. Abortion is an emergency contraceptive method.** |  |  |  |
| **35. Family planning is mainly women’s responsibility.** |  |  |  |
| **36. Modern methods are more efficient than traditional methods.** |  |  |  |

**THANK YOU FOR YOUR PARTICIPATION.**
