# Supplementary material for: The knowledge and perceptions of the first year medical students of an International University on family planning and emergency contraception in Nicosia (TRNC)
Source: BMC Womens Health. 2018 Sep 15;18:149. doi: 10.1186/s12905-018-0641-x (PMC6139172; doi:10.1186/s12905-018-0641-x)
Supplement: Supplementary file 2 — Turkish questionnaire. (DOCX 30 kb) [file 12905_2018_641_MOESM2_ESM.docx]

**YAKIN DOĞU ÜNİVERSİTESİ TIP FAKÜLTESİ DÖNEM-1 ÖĞRENCİLERİNİN AİLE PLANLAMASI VE ACİL KONTRASEPSİYON YÖNTEMLERİ HAKKINDAKİ BİLGİ VE DAVRANIŞLARI**

ANKET FORMU

Anket No: ……

Lütfen anket formuna **isminizi yazmayınız**. **Araştırmaya katılmayı Kabul ediyorum. □**

**Kabul etmiyorum. □**

**A. SOSYODEMOGRAFİK ÖZELLİKLER İLE İLGİLİ SORULAR**

**1. Kaç yaşındasınız?** (**Bitirmiş olduğunuz yaşınızı yazınız**) …….

**2. Cinsiyetiniz nedir?** 1) Kadın 2) Erkek

**3. Hangi ülkenin vatandaşı olduğunuzu belirtiniz.** ………………………………

**4. 12 yaşına kadar en uzun süre yaşadığınız ülke neresidir?**

1) KKTC 2) Türkiye 3) İngiltere 4) Suriye

5) Diğer (Neresi olduğunu belirtiniz) ……..............

**5. Medeni durumunuz nedir?**

1) Evli 2) Bekar 3) Diğer ……..

**6. Annenizin öğrenim düzeyi nedir?**

1) Okuryazar değil 2) İlköğretim 3) Ortaöğretim 4) Lise

5) Yüksekokul/Üniversite

**7. Babanızın öğrenim düzeyi nedir?**

1) Okuryazar değil 2) İlköğretim 3) Ortaöğretim 4) Lise

5) Yüksek okul/Üniversite

**8. Şu anda kiminle yaşıyorsunuz?**

1) Ailem ile birlikte 2) Yalnız 3) Partnerimle birlikte

4) Arkadaşlarımla birlikte 5) Diğer (Kiminle yaşadığınızı belirtiniz ……………….)

**B. AİLE PLANLAMASI YÖNTEMLERİ HAKKINDA BİLGİ DÜZEYİ VE DAVRANIŞ SORULARI**

**9. Aşağıdaki tanımlardan hangisi aile planlamasını en iyi tanımlamaktadır?**

1) Aile planlaması bireylerin çocuk sahibi olmalarını engellemektir.

2) Aile planlaması, ailenin maddi ve manevi kaynaklarını planlamaktır.

3) Aile planlaması, bireylerin istedikleri zaman istedikleri sayıda çocuk sahibi olmaları

demektir.

4)Aile planlaması; çiftlerin en az bir çocuk sahibi olmasını sağlamaktır.

5)Aile planlaması; infertil çiftlerin tüp bebekle çocuk sahibi olmalarını sağlamaktır.

**10. Aile planlaması/acil kontrasepsiyon hakkında daha önce bilgi edindiniz mi?**

1) Evet

2) Hayır **(12. soruya geçiniz.)**

**11. Aile planlaması yöntemleri ile ilgili bilgiyi nereden aldınız? (Birden fazla seçenek işaretleyebilirsiniz.)**

1) Sağlık personeli

2) Eczane

3) İnternet

4) Basın- Yayın (Dergi/gazete/kitap)

5) Arkadaş

6) Anne, baba, akraba

7) Okul

8) TV

9) Diğer (Belirtiniz:…...………

**12. Aşağıdaki kontrasepsiyon yöntemlerinden hangileri modern (M), hangileri geleneksel (G) yöntemdir? (M ve G olarak işaretleyiniz.)**

1. Oral kontraseptif/Hap
2. Emzirme
3. Spermisit
4. Geri çekme
5. Östrojen ve progesteron enjeksiyonu
6. Kombine patch (yama)
7. Kombine vajinal halka
8. İmplant
9. Rahim İçi Araçlar
10. Takvim metodu
11. Kadın sterilizasyonu(tüp ligasyonu)
12. Erkek kanallarının bağlanması
13. Erkek Kondomu
14. Kadın Kondomu
15. Diyafram
16. Servikal Başlık

**13. Daha önce herhangi bir kontraseptif yöntem kullandınız mı?**

1) Evet

2) Hayır **(15. soruya geçiniz.)**

**14. Hangi kontraseptif yöntemi kullandığınızı belirtiniz. (Birden fazla seçenek işaretlayabilirsiniz.)**

**…………………………………………………………………………………………**

1) Hap 2) Kondom 3) RİA 4) Enjeksiyonlar

5) Tüp ligasyon ( kadının tüplerini bağlatması) 6) Vazektomi (Erkeğin kanallarını bağlatması)

7) Takvim yöntemi 8) Geri çekme

9) Diğer (Belirtiniz)…………………………

**C.ACİL KONTRASEPSİYON HAKKINDA BİLGİ SORULARI**

**15. Sizce acil kontrasepsiyon nedir?**

1)Her gün kullanılması gereken doğum kontrol yöntemi.

2)Korunmasız cinsel ilişki sonrası kullanılan doğum kontrol yöntemi.

3)Cinsel yolla bulaşan enfeksiyonları engellemek için kullanılan bir yöntem.

**16. Acil kontrasepsiyon hangi amaçla kullanılır?**

1) Gebeliği sonlandırmak için kullanılır.

2) İmplantasyonu engelleyerek gebeliği önlemek amacıyla kullanır.

3) Cinsel yolla bulaşan hastalıkları önlemek için kullanılır.

**17. Aşağıdakilerden acil kontrasepsiyon yöntemi olduğunu düşündüklerinizi işaretleyiniz.**

1. Kondom 2. Ertesi gün hapı 3. Kombine oral hap

4. Rahim içi araç 5. Implant 6. Enjektabl kontraseptif 7. Geri çekme

8.Kürtaj

**18. Acil kontrasepsiyon hangi durumlarda kullanılabilir? (Birden fazla seçenek işaretleyebilirsiniz)**

1) Korunmasız cinsel ilişki

2) Kontraseptif yöntemin başarısız olması

3) Tecavüz

4) İstenmeyen gebeliklerde

5) Aile planlaması yöntemi olarak

**19. Acil kontrasepsiyonun en etkili olarak kullanıldığı dönem hangisidir?**

1) Cinsel ilişki öncesi

2) Korunmasız cinsel ilişki sonrası 24 saat içinde

3) Korunmasız cinsel ilişki sonrası 120 saat (5 gün) içinde

4) Bir sonraki adet kanamasına kadar

**20.Ertesi gün hapı nereden temin edilir? Birden fazla şık işaretleyebilirsiniz.**

1)Aile sağlığı merkezleri 2) Eczaneden reçete ile 3) Eczaneden reçetesiz

**21.Rahim içi araç nerede takılır?**

**………………………………………………………………………….**

**22. Acil kontrasepsiyonun sağlığı olumsuz etkileyecek yan etkileri var mıdır? Varsa belirtiniz.**

**23.Bugüne kadar yakın çevrenizden birinin bu hizmete gereksinimi oldu mu?**

1) Evet 2) Hayır

Evetse ne yaptı? …………………………………………………………………

**24. Acil kontrasepsiyon hapı nedir? Kısaca anlatınız. ……………………………**

**25. Bugüne kadar hiç cinsel ilişki deneyiminiz oldu mu?**

1) Evet,oldu.

2) Hayır,olmadı. **(31. soruya geçiniz.)**

**26. İlk cinsel ilişki deneyiminizi kaç yaşında yaşadınız? Belirtiniz …………….**

**27. İlk ilşkinizde siz ya da partneriniz gebeliği önleyici bir yöntem kullandınız mı?**

1) Evet (Yöntemi belirtiniz: ………………………………………….)

2) Hayır

**28. Halen düzenli bir cinsel yaşantınız var mı?**

1) Evet

2) Hayır

**29. Daha önce siz ya da partneriniz acil kontraseptif bir yöntem kullandınız mı?**

1) Evet

2) Hayır (31. soruya geçiniz )

**30. Siz ya da partneriniz acil kontraseptif yöntem kullandıysanız, ne kullandığınızı belirtiniz: …………………………………………….**

**D. AŞAĞIDAKİ SORULARDA DOĞRU (D), YANLIŞ (Y) YA DA BİLMİYORUM (B) SEÇENEKLERİNİN ALTINI İŞARETLEYİNİZ**

|  | D | Y | B |
| --- | --- | --- | --- |
| **31. Acil kontrasepsiyon korunmasız cinsel ilişki sıklığını artırır.** |  |  |  |
| **32. Acil kontrasepsiyon istenmeyen gebelik sonucu kürtaj sıklığını azaltır.** |  |  |  |
| **33. Kişiler eczaneden ertesi gün hapı alırken utanırlar.** |  |  |  |
| **34. Kürtaj bir acil kontrasepsiyon yöntemidir.** |  |  |  |
| **35. Aile planlaması esas olarak kadının sorumluluğudur** |  |  |  |
| **36. Modern metodlar geleneksel metodlardan daha etkilidir.** |  |  |  |

**KATKILARINIZ İÇİN TEŞEKKÜR EDERİZ.**
